# *Mycobacterium tuberculosis* ClpP1 and ClpP2 Function Together in Protein Degradation and Are Required for Viability *in vitro* and During Infection

**DOI:** 10.1371/journal.ppat.1002511

**Published:** 2012-02-16

**Authors:** Ravikiran M. Raju, Meera Unnikrishnan, Daniel H. F. Rubin, Vidhya Krishnamoorthy, Olga Kandror, Tatos N. Akopian, Alfred L. Goldberg, Eric J. Rubin

**Affiliations:** 1 Department of Immunology and Infectious Diseases, Harvard School of Public Health, Boston, Massachusetts, United States of America; 2 Department of Cell Biology, Harvard Medical School, Boston, Massachusetts, United States of America; Weill Medical College of Cornell University, United States of America

## Abstract

In most bacteria, Clp protease is a conserved, non-essential serine protease that regulates the response to various stresses. Mycobacteria, including *Mycobacterium tuberculosis* (Mtb) and *Mycobacterium smegmatis*, unlike most well studied prokaryotes, encode two ClpP homologs, ClpP1 and ClpP2, in a single operon. Here we demonstrate that the two proteins form a mixed complex (ClpP1P2) in mycobacteria. Using two different approaches, promoter replacement, and a novel system of inducible protein degradation, leading to inducible expression of *clpP1* and *clpP2*, we demonstrate that both genes are essential for growth and that a marked depletion of either one results in rapid bacterial death. ClpP1P2 protease appears important in degrading missense and prematurely terminated peptides, as partial depletion of ClpP2 reduced growth specifically in the presence of antibiotics that increase errors in translation. We further show that the ClpP1P2 protease is required for the degradation of proteins tagged with the SsrA motif, a tag co-translationally added to incomplete protein products. Using active site mutants of ClpP1 and ClpP2, we show that the activity of each subunit is required for proteolysis, for normal growth of Mtb *in vitro* and during infection of mice. These observations suggest that the Clp protease plays an unusual and essential role in Mtb and may serve as an ideal target for antimycobacterial therapy.

## Author Summary

Due to the significant and rapid rise in multidrug resistant *Mycobacterium tuberculosis* (Mtb), there is an urgent need to validate novel drug targets for the treatment of tuberculosis. Here, we show that Clp protease is an ideal potential target. Mtb encodes two ClpP genes, ClpP1 and ClpP2, which associate together to form a single proteolytic complex, referred to as ClpP1P2. Both proteins are required for growth *in* vitro and in a mouse model of infection. Depletion of either protein results in rapid death of the bacteria. Interestingly, this is rare among bacteria, most of which have only one ClpP gene that is dispensable for normal growth. We also show that Clp protease plays an important quality control role by clearing abnormally produced proteins. As known antimycobacterial therapeutics increase errors in protein synthesis, inhibitors of ClpP1P2 protease in Mtb may prove synergistic with already existing agents.

## Introduction

Intracellular protein degradation is critical for maintaining cellular homeostasis through protein quality control and regulation of numerous biological pathways [1], [2]. In eukaryotes, the ubiquitin-proteasome pathway constitutes the predominant degradation pathway [3]. Most prokaryotes, however, possess a variety of ATP-dependent serine protease complexes, such as Lon and Clp protease [4], and some actinomycetes and archaea contain proteasomes, which are threonine proteases. Interestingly, *Mycobacterium tuberculosis* (Mtb) encodes both a proteasome and Clp protease. While recent work has explored the role of the Mtb proteasome [5]–[7], little is known about mycobacterial Clp protease. This serine protease was first discovered and is best characterized in *Escherichia coli* [8], [9]. The Clp proteolytic complex is formed by the association of proteolytic subunits, ClpP, with ATPase adapters, ClpX or ClpA in Gram-negative organisms and ClpX or ClpC in Gram-positive organisms. *E. coli* ClpP is a tetradecamer composed of two stacked heptameric rings of identical ClpP subunits that form an internal proteolytic chamber [10]. This core associates with distinct hexameric ATPase adapters, ClpX and ClpC1 in mycobacteria, which provide substrate specificity and catalyze ATP-dependent unfolding of globular proteins [11], [12]. In *E. coli*, the ClpXP protease is involved in the regulation of the DNA damage response and degradation of SsrA-tagged peptides stalled on the ribosome [13], [14]. Clp proteolytic enzymes are also required for full virulence in several pathogenic organisms, including *Listeria monocytogenes* where the protease is required for the production of α-listeriolysin [15], [16]. In most bacteria including *E. coli*, Clp protease is dispensable for normal growth, and in fact, until recently, the only organism in which *clpP* has been found to be essential is *Caulobacter crescentus*, where Clp degrades CtrA, an inhibitor of cell cycle progression [17].

Unlike most bacteria, which have a single ClpP subunit, the genome of Mtb encodes two closely related ClpP homologs, *clpP1* and *clpP2*, in a single operon. A transposon-based mutagenesis screen for essential genes in Mtb predicted that ClpP2 and the ATPase adapters ClpC1 and ClpX, were required for normal growth [18] while a recent publication has shown that ClpP1 is essential [19]. Here, we show that both ClpP1 and ClpP2 are required for growth, and that their activity is important for the removal of abnormal proteins. Our data suggest that ClpP1 and ClpP2 assemble to form a single proteolytic complex, referred to as ClpP1P2, that is required for normal growth *in vitro* and during infection. In related studies, we have found that although pure ClpP1 and ClpP2 by themselves form tetradecamers, they are inactive. However, in the presence of low molecular weight activators they reassociate to form a mixed tetradecamer, ClpP1P2, which is capable of proteolysis (Akopian et. al., manuscript submitted). The unusual properties of this heteromeric complex, the absence of such an enzyme in the eukaryotic cytoplasm, and the essentiality of both subunits make ClpP1P2 protease an attractive target for novel therapeutic development for the treatment of tuberculosis.

## Results

### ClpP1 and ClpP2 subunits interact to form a single proteolytic complex

**Figure 1:**
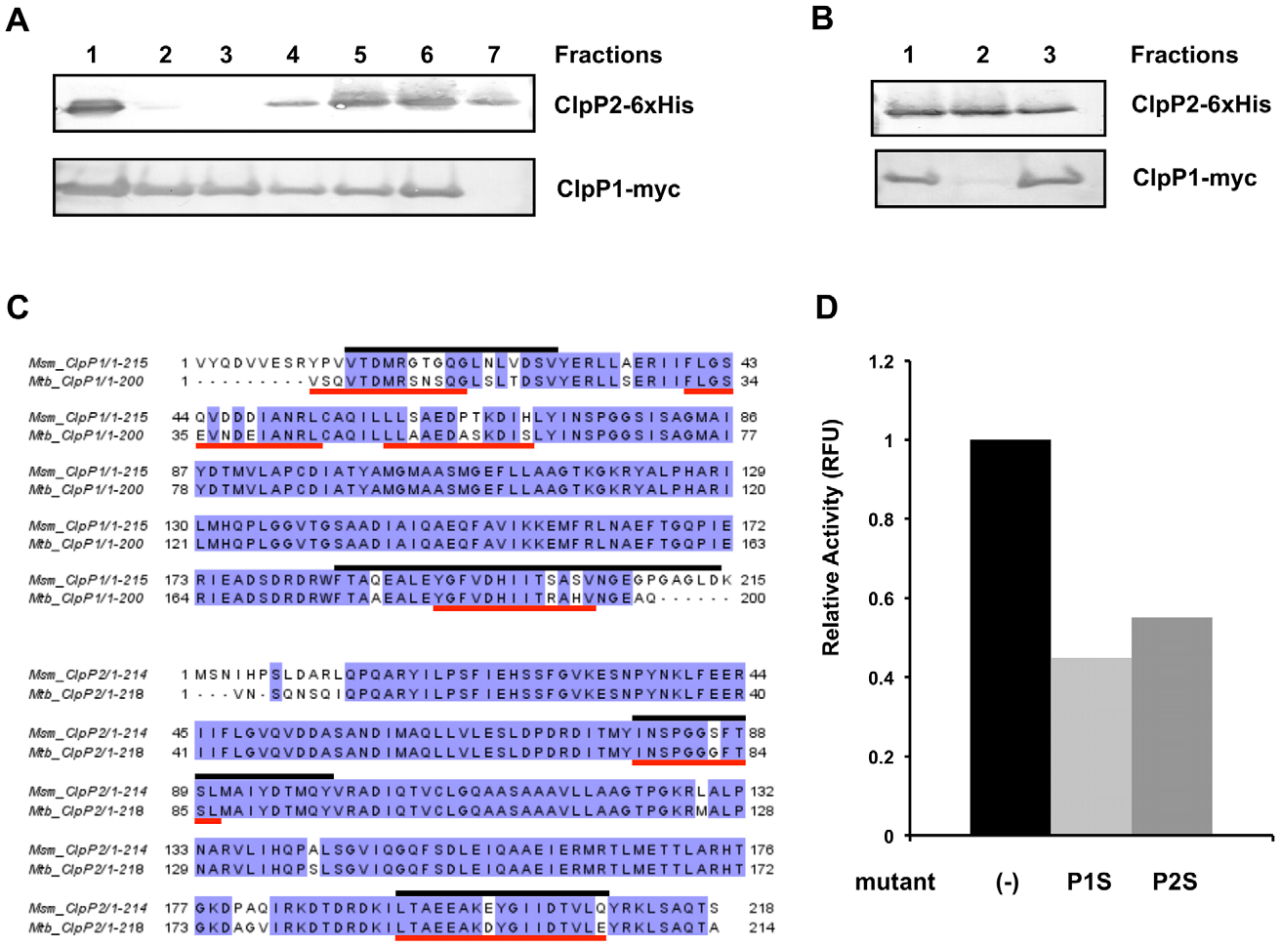
Mtb ClpP1 and ClpP2 interact, forming a multi-component protease, and share substantial similarity with ClpP1 and ClpP2 homologs in Msm. (A) C-terminally myc-tagged Mtb ClpP1 and 6×His-tagged Mtb ClpP2 were expressed in Msm. Lysate (lane 1) was prepared and loaded onto a Ni-column. After washing with PBS (lanes 2,3), Ni-bound material was eluted with 50 mM (lane 4), 100 mM (lane 5), 250 mM (lane 6, 7) of imidazole in PBS, and analyzed by immunoblotting using anti α-myc and α-6×His antibodies. (B) Fraction 6 from (A) was applied to an anti-myc column (lane 1). The flow through (lane 2), and bound material (lane 3) were analyzed by immunoblot with α-myc and α-His antibodies. Bound material was released from the anti-myc agarose beads by boiling in Laemmli buffer after washing with PBS. (C) Bands representing ClpP1 and ClpP2 from (B) were sequenced by MS/MS revealing the presence of both Mtb and Msm homologs. Msm specific peptides are indicated by black lines, those specific to Mtb are indicated by red lines. (D) Cleavage of fluorescent peptide Z-Gly-Gly-Leu-AMC was measured in the presence of 1 µg ClpP1, 1 µg Clp2, and the activating peptide Z-Leu-Leu (see accompanying paper). Addition of 5 µg of catalytically inactive mutants of either ClpP1 (ClpP1S) or ClpP2 (ClpP2S) markedly inhibited cleavage by the ClpP1P2 protease. Results graphed are a representative sample of results obtained.

Mycobacterial genomes contain two homologous ClpP protease genes, *clpP1* and *clpP2*, arranged in a putative operon. To investigate whether the two proteins may function together in a complex, we co-expressed Mtb *clpP1* and *clpP2*, each containing a different C terminal epitope tag, in *Mycobacterium smegmatis* (Msm). We used affinity chromatography with nickel resin to isolate 6×-His tagged Mtb ClpP2 together with associated proteins from the Msm cell lysate. As shown in **Figure 1A**, a fraction of the c-myc tagged ClpP1 bound to the Ni column and co-eluted with ClpP2. To verify that ClpP1 and ClpP2 co-eluted from the Ni column may be associated in a complex, we applied the fraction from the Ni column containing both proteins to an anti-c-myc agarose column and analyzed by SDS PAGE. **Figure 1B** shows that a large fraction of the ClpP2 was associated with ClpP1. Incidentally, expression of the Mtb proteins in Msm also led to the co-isolation of Msm ClpP1 and ClpP2, as shown by tandem mass spectrometry of the purified complex. In each case, peptides present uniquely in Mtb or Msm ClpP1 and ClpP2 were detected (**Figure 1C**).

If ClpP1 and ClpP2 do in fact associate to form a single proteolytic core, we reasoned that mutations blocking the catalytic activity of one subunit might reduce that activity of the enzyme. We identified likely active site residues of ClpP1 and ClpP2 by mapping the Mtb proteins onto *E. coli* ClpP and locating the catalytic triad of Asp-His-Ser, which is characteristic of serine proteases. In both cases, the serine likely to be responsible for nucleophillic attack was replaced by an alanine (ClpP1 S98A and ClpP2 S110A). To analyze the effects of these mutations, we expressed and purified 6×His-tagged forms of each protein,and assayed their effect on the enzymatic activity of the wild type ClpP1P2 in an *in vitro* peptidase assay (Akopian et. al., manuscript submitted). Enzyme activity of the reconstituted ClpP1P2 complex was quantified using cleavage of the fluorescence reporter, Z-Gly-Gly-Leu-AMC. As seen in **Figure 1D**, addition of an excess of mutated ClpP1 or ClpP2 to the active wild type ClpP1P2 complex inhibited proteolytic cleavage of a fluorescent peptide substrate, presumably by replacing the wild type subunits. These results suggest that the ClpP1 and ClpP2 subunits interact to form a single proteolytic complex *in vitro*, that each active site is important for activity, and that these mutations can be used as dominant negative inhibitors.

### Both ClpP1 and ClpP2 are required for normal growth *in vitro*

**Figure 2:**
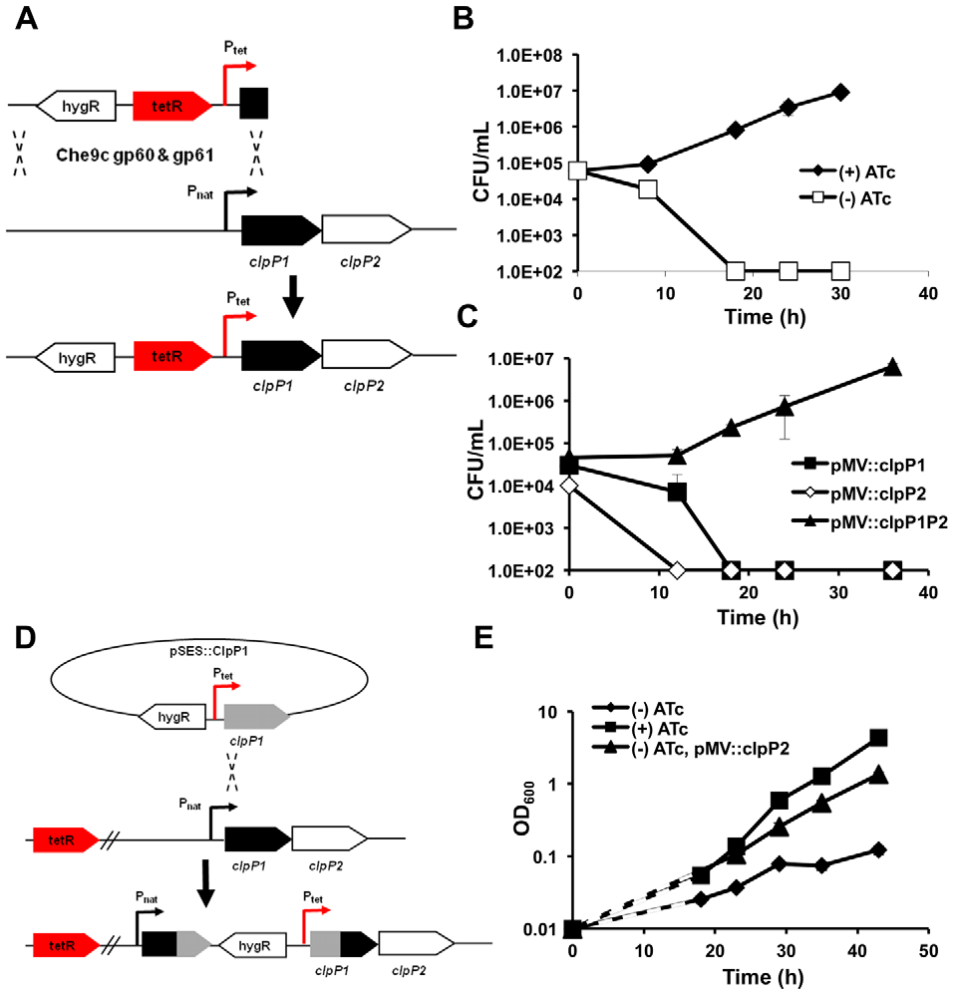
Both ClpP1 and ClpP2 are essential for normal growth in mycobacteria. (A) Schematic representation of mycobacterial recombineering, employed to replace the endogenous promoter of the clpP1P2 operon with a ATc-inducible promoter (Msm strain ptet_clpP1P2). (B) Growth curves of Msm ptet_clpP1P2 in the presence (50 ng/mL) or absence of inducer ATc. Data are represented as mean CFU/mL +/− standard deviation. (C) Growth curves of Msm ptet_clpP1P2 complemented with clpP1, clpP2 or both clpP1 and clpP2 in the absence of inducer ATc. Data are represented as mean CFU/mL +/− standard deviation. (D) Schematic representation of genetic strategy used to create a tetracycline inducible conditional Msm ClpP2 mutant (Msm strain ptet_ClpP2) (E) Growth curves of Msm ptet_clpP2 in the presence (50 ng/mL) or absence of inducer ATc. Msm ptet_clpP2 was also complemented with clpP2 in the absence of ATc. Data are represented as mean OD_600_ +/− standard deviation. Dashed lines represent assumed growth rates until first measured growth point.

We employed three complementary strategies to determine if ClpP1 and ClpP2 are required for normal growth in mycobacteria. First, using mycobacterial recombineering [20], we replaced the endogenous promoter of *clpP1* and *clpP2* in Msm with a tetracycline-inducible promoter (**Figure 2A** **, Figure S1**). Introduction of a tetracycline repressor resulted in a strain (ptet_clpP1P2) that could only be maintained in the presence of the inducer anhydrotetracycline (ATc) (**Figure 2B**). In the absence of this compound, growth did not occur, but could be restored by the presence of an episomal plasmid containing both *clpP1* and *clpP2*. Plasmids expressing only *clpP1* or c*lpP2* alone could not rescue growth and depletion of either subunit resulted in bacterial death (**Figure 2C**). Since complementation was conducted with Mtb homologs and subunits from different species associate into a functional tetradecamer, the ClpP1P2 complex is likely very similar in Msm and Mtb. Furthermore, active site mutants of either ClpP1 or ClpP2 were unable to complement ptet_clpP1P2 in the absence of ATc, suggesting that the activity of both subunits were required for normal growth (data not shown).

Second, we inserted a tetracycline inducible promoter upstream of the *clpP1P2* operon via homologous recombination in Msm creating a strain in which *clpP2* was inducibly expressed (**Figure 2D**), and *clpP1* was under the control of its native promoter (ptet_clpP2). In accord with the previous findings, the growth of this strain was dramatically inhibited in the absence of ATc (**Figure 2E**).

**Figure 3:**
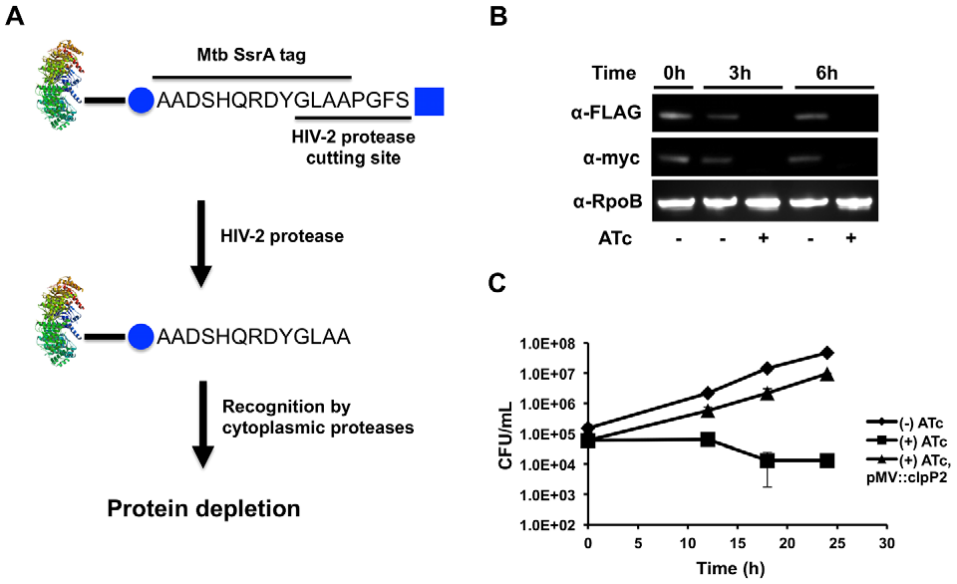
Inducible protein degradation demonstrates requirement of ClpP2 for normal growth. (A) Schematic representation of the inducible degradation system used to inducibly deplete ClpP2 (Msm strain clpP2_ID). Induction of HIV-2 protease with ATc leads to cleavage of the HIV-2 protease cutting site and exposure of a SsrA tag on the tagged protein. Cleavage by HIV-2 protease and subsequent degradation can be tracked via the FLAG (square) and c-myc (circle) epitope tags, respectively, included on the inducible degradation tag. (B) Degradation of ClpP2 in clpP2_ID was tracked by Western in the absence or presence of inducer ATc. Blots were probed α-FLAG (loss indicates HIV-2 protease cleavage), α-myc (loss indicates target degradation), and α-RpoB (loading control). (C) Growth curves of Msm clpP2_ID in the absence or presence (50 ng/mL) of inducer ATc. Msm clpP2_ID was also complemented with clpP2 in the presence of ATc. Data are represented as mean CFU/mL +/− standard deviation.

Third, we used a system of inducible protein degradation recently developed in Msm (**Figure 3A**) [21]. Briefly, we employed mycobacterial recombineering to add an inducible degradation (ID) tag to the C-terminus of ClpP2 (clpP2_ID). Upon cleavage of the tag by a tetracycline inducible HIV-2 protease, an SsrA sequence is revealed on the substrate that directs degradation of the protein. By inserting epitope tags C-terminally to the HIV-2 protease recognition motif (FLAG) and N-terminally to the SsrA tag (c-myc), we were able to monitor the amount of ClpP2 by immunoblotting. As shown in **Figure 3B**, induction of HIV-2 protease resulted in degradation of the majority of ClpP2 and inhibited bacterial growth (**Figure 3C**). Using this system, we did not observe cell death, perhaps due to incomplete inhibition, as would be expected for a system where the protease targets itself. Loss of ClpP2, as measured by immunoblotting, was rapid and reached near completion within hours. Furthermore, the growth defect was complemented by expression of Mtb *clpP2* using a constitutively active promoter. A similar approach with ClpP1 was unsuccessful as extended C-terminal tagging was not tolerated, and the ID tag was indiscriminately cleaved. Collectively, these results confirm that both ClpP1 and ClpP2 are required for normal growth in mycobacteria, presumably because they function together in the ClpP1P2 complex.

### Mycobacterial Clp protease plays a role in protein quality control

**Figure 4:**
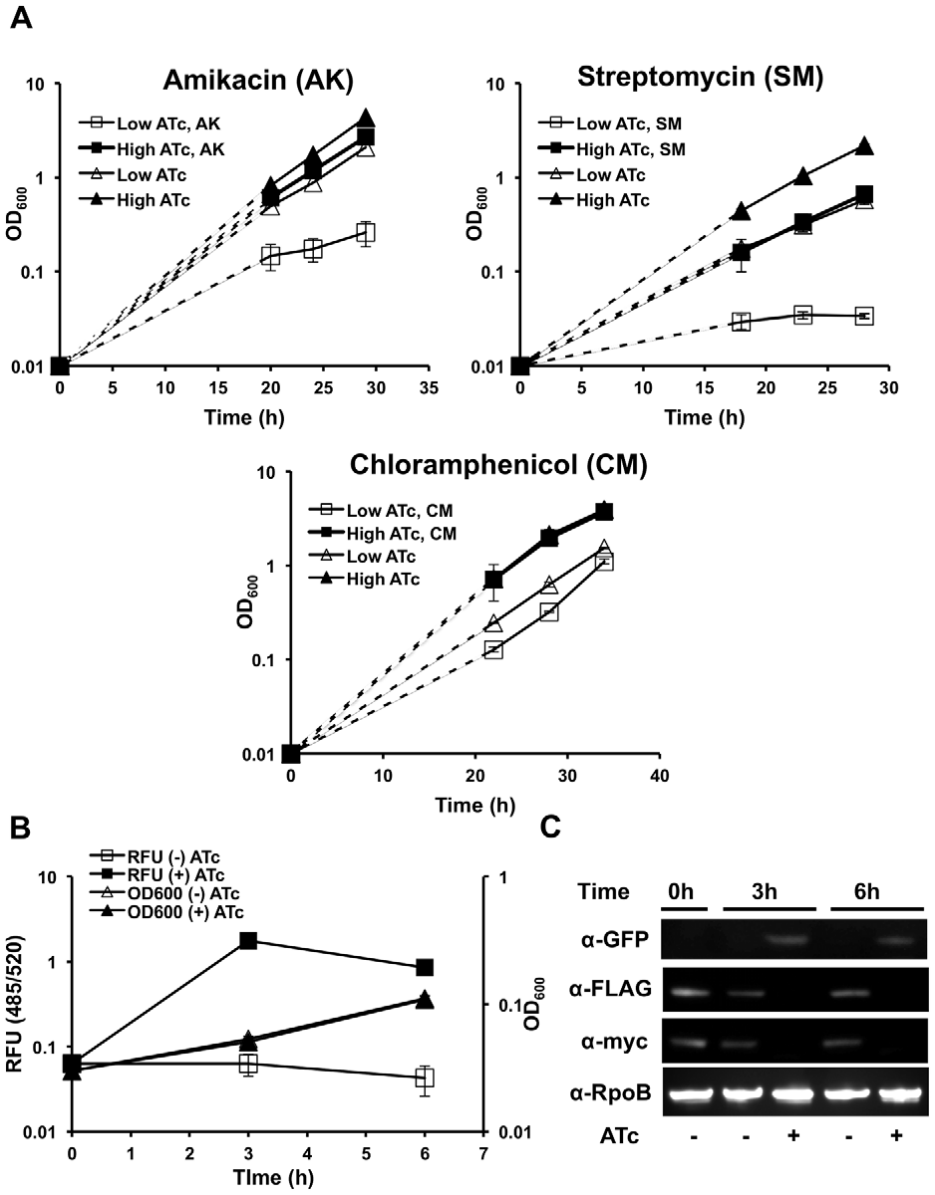
Clp protease is required for degradation of abnormal proteins and SsrA-tagged proteins in mycobacteria. (A) Growth curves of Msm ptet_clpP2 in growth medium containing low (1 ng/mL) or high (100 ng/mL) concentrations of inducer ATc, in the presence of either no drug, or amikacin (top left, 0.03 µg/mL), streptomycin (top right, 0.125 µg/mL), and chloramphenicol (bottom, 7.5 µg/mL). Data are represented as mean OD_600_ +/− standard deviation. Dashed lines represent assumed growth rates until first measured growth point. (B) Increase in fluorescence (RFU, 485/520) and initial growth curve (OD_600_) of Msm clpP2_ID expressing the fusion construct GFP-SsrA on a constitutively expressing plasmid, in the presence and absence of inducer, ATc. Data are represented as mean RFU or OD_600_ +/− standard deviation. (C) Depletion of ClpP2 and increase in GFP-SsrA in Msm clpP2_ID expressing the fusion construct GFP-SsrA on a constitutively expressing plasmid was tracked by immunoblot. Blots were probed α-GFP, α-myc, α-FLAG, and α-RpoB (loading control).

In other bacteria, Clp plays a role in degrading abnormal proteins such as SsrA-tagged peptides that stall on the ribosome [22]. To determine the importance of ClpP1P2 protease in the degradation of misfolded proteins, we used antibiotics that alter protein synthesis in distinct ways including chloramphenicol, which blocks protein elongation without increasing mistranslation rates [23], and streptomycin and amikacin, which induce translational errors resulting in missesnse or prematurely-terminated polypeptides [24]. We found that the strain ptet_ClpP2, in which *clpP2* expression is regulated by anhydrotetracycline, grows well in low or high concentrations of ATc, 1 to 100 ng/mL (**Figure 4A**). Treatment with sublethal concentrations of chloramphenicol resulted in no difference in viability between bacteria maintained on low or high concentrations of ATc (**Figure 4A** **, bottom**). In contrast, sub-MIC concentrations of the aminoglycosides streptomycin and amikacin significantly inhibited the growth of strains incubated in low concentrations of ATc, while they had no effect on growth of the strain maintained in high concentrations of ATc (**Figure 4A** **, top**). Together, these results suggest that ClpP1P2 protease protects against error-prone translation by catalyzing the degradation of misfolded proteins.

To specifically assess whether ClpP1P2 is responsible for the removal of SsrA-tagged proteins in mycobacteria, we fused the mycobacterial SsrA-tag to the C-terminus of GFP (GFP-SsrA) and expressed the construct constitutively on an episomal plasmid. This construct was introduced into the strain clpP2_ID, in which ClpP2 degradation was regulated. In the presence of ClpP2 (and in wild type cells), there was no detectable GFP-SsrA. However, upon depletion of ClpP2, there was substantial accumulation of GFP-SsrA, as measured by both fluorescence and immunoblot analysis after four hours (**Figure 4B, 4C**). Quantitative PCR showed that the rise of GFP-SsrA was not due to transcriptional activation of the gene (**Figure S2**). GFP lacking the SsrA tag is present at similar levels in all strains (data not shown). Because we cannot detect GFP-SsrA in the presence of Clp activity, we were unable to accurately measure changes in protein stability. However, the rate of accumulation of GFP-SsrA was consistent with the time course of ClpP2 depletion, which occurred over six hours, as shown by immunoblotting. Thus, functional ClpP1P2 protease is vital for the rapid clearance of SsrA-tagged substrates in mycobacteria.

### Functional Clp protease is required for growth of Mtb *in vitro* and during infection

**Figure 5:**
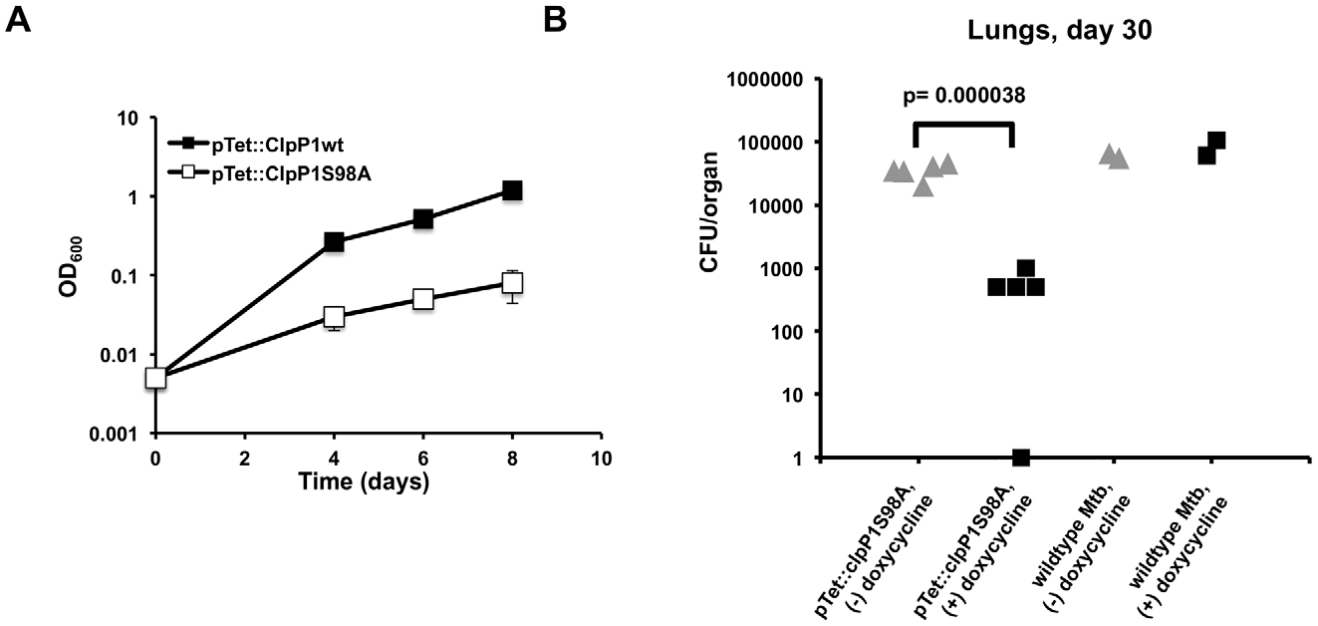
A catalytically inactive ClpP allele inhibit Mtb growth *in vitro and during infection*. (A) Growth curves for Mtb overexpressing wild type ClpP1 or ClpP1 S98A via an ATc-inducible expression vector. Data are represented as mean OD_600_ +/− standard deviation. Dashed lines represent assumed growth rates until first measured growth point. (B) Growth of Mtb containing a doxycycline-inducible plasmid expressing the mutant allele ClpP1 S98A in lungs of C57BL/6 mice 30 days post aerosol infection. Mice were infected via aerosol with a 3∶1 mixture of mutant and wild type bacteria. Mice were fed either with chow containing (dark squares, N = 5 mice) or lacking (gray triangles, N = 5 mice) the inducer doxycycline. As a control, wild type Mtb was co-infected, and representative CFU/organs for the control are represented (right). Each point represents calculated total CFU/organ for each mouse. Not all mice received enough wild type bacteria to quantitate.

As shown above, catalytically inactive forms of ClpP1 and ClpP2 inhibit proteolysis by the wild type enzyme, possibly by replacement of wild type subunits with inactive ones. To assess whether ClpP1P2 activity is required for the growth of Mtb, we expressed a catalytically inactive form of Mtb *clpP1*, *clpP1 S98A*, on a tetracycline-inducible plasmid in wild type Mtb. Addition of ATc led to expression of the catalytically inactive mutant protein and resulted in a significant inhibition of growth (**Figure 5A**) while overexpression of wild type Mtb *clpP1* had no effect.

To determine if the dominant negative mutant of ClpP1 affected ClpP1P2 function during infection, we infected mice with a 3∶1 mixture of Mtb expressing *clpP1 S98A* on a hygromycin-resistant doxycycline inducible plasmid and wild type Mtb (containing a kanamycin-resistant control vector). Mice were fed either normal chow or chow infused with the inducer doxycycline. Growth of Mtb was monitored by assessing CFU in lung tissue at day 30 post-infection. While there were no differences in the growth of wild type Mtb between treated and untreated mice, expression of the active site mutant significantly inhibited growth (**Figure 5B**). Our results suggest that functional ClpP1P2 protease is required for the growth of Mtb both *in vitro* and during infection.

## Discussion

We find that the mycobacterial ClpP1P2 protease has two unusual properties that distinguishes it from other members of the prokaryotic ClpP family. First, the protease consists of distinct types of subunits, each of which is required for full activity of a single proteolytic complex. While other species do encode multiple ClpP subunits, two different proteolytic subunits forming a single protease has not been documented. Second, unlike in most bacteria that have been studied, ClpP1P2 activity is absolutely required for normal growth. This requirement is particularly striking as mycobacteria contain several cytoplasmic ATP-dependent proteolytic complexes, including FtsH, and the proteasome [7], [25], [26]. Clearly, the mycobacterial ClpP1P2 proteolytic core has unique roles that are important for viability.

The ClpP proteases that have been characterized biochemically in other bacteria and mitochondria are tetradecameric complexes containing a single type of proteolytic subunit. In mycobacteria, however, two different protein species contribute to protease activity. Although Mtb ClpP1 forms a tetradecameric complex, a crystal structure of Mtb ClpP1 lacks appropriate active site geometry to support proteolysis [27]. The presence of two ClpP subunits with distinct substrate preferences may facilitate an expansion of the peptide specificity of the complex, much like the eukaryotic proteasome. Interestingly, the Mtb proteasome is composed of a single type of subunit, and the presence of distinct subunits comprising a single proteolytic core is rare among prokaryotes.

There is at least one example of an essential role for ClpP. In *Caulobacter crescentus*, ClpXP degrades CtrA, a protein that normally inhibits cell cycle progression during cellular replication [28]. In this case, a single protein target is responsible for the essentiality of the enzyme. ClpP1P2 protease might play a similar role in mycobacteria. While this may be true, screens for essential proteins in mycobacteria suggest that, in addition to the clpP1 and clpP2, multiple Clp-associated ATPase adapters (clpX and clpC1) are also essential [18]. The requirement of multiple adapters makes it possible that accumulation of multiple protein substrates contribute to the poor growth phenotype observed on depleting the ClpP1 and ClpP2 subunits in mycobacteria.

ClpP1P2 might be important for other reasons. As shown here, ClpP1P2 protease is required for the clearance of SsrA-tagged proteins. These tagged polypeptides are generated under conditions when protein synthesis is stalled and are required for ribosome release. In the absence of ClpP1P2-mediated proteolysis, protein synthesis might eventually be inhibited. In addition, ClpP1P2 protease is necessary for degrading abnormal proteins, such as those produced in the presence of certain antibiotics. Accumulation of such non-functional misfolded proteins might result in cellular stress in the absence of an effective system for their removal [29]. Clearance of damaged proteins might be particularly important in Mtb during infection, when cells are exposed to multiple oxidative and nitrosoative radicals that can induce protein damage. In fact, a transcriptional activator of the *clpP1P2* operon, clgR, is critically activated upon reaeration of hypoxic Mtb and during Mtb growth within the macrophage [30], [31]. Degradation of pre-existing proteins during such stressful transitions may be the initial event that triggers adaptation and facilitates the bacterium's ability to handle a wide array of environmental challenges. Using a dominant negative overexpression mutant in Mtb, we have confirmed that optimal Clp proteolytic activity is required for growth during infection.

The essential nature of ClpP1P2 protease makes it an attractive target for antibiotic development, particularly because the proteases as a class are druggable enzymes and have already been validated as therapeutic targets in the treatment of HIV, hepatitis, and cancer [32]. In organisms where ClpP is not essential, uncontrolled activation of ClpP activity can be toxic. For example, in *E. coli*, acyldepsipeptide compounds reorganize the ClpP proteolytic core, causing dissociation from ATPase adapters, and indiscriminate protein degradation [33]. Compounds that produce a similar effect should result in toxicity in a broad range of organisms. In fact, it was recently discovered that the natural product cyclomarin kills Mtb by targeting the ClpC1 ATPase and presumably increasing Clp-mediated proteolysis, as demonstrated in a whole cell fluorescence-based assay [34]. In mycobacteria, where ClpP1P2 protease activity is required and depletion of either subunit is bactericidal, either non-specific activation or inhibition could effectively limit bacterial growth. An example of a ClpP inhibitor with potential therapeutic activity already exists. In *S. aureus*, beta-lactones have been found to inhibit Clp protease activity and decrease the virulence of the organism [35]. Additionally, the synergistic nature of ClpP1P2 protease depletion with aminoglycosides, a class of drugs already used to treat tuberculosis, points to a potential combination therapy against Mtb. As ClpP1P2 protease is most likely involved in preventing the accumulation of misfolded proteins and the degradation of critical endogenous regulatory proteins, small molecule modulators of ClpP1P2 activity would target a critical aspect of Mtb physiology, and might prove useful in the face of growing multi-drug resistance in one of the world's most successful pathogens.

## Materials and Methods

### Ethics statement

The animal experiments were preformed with protocols approved by the Harvard Medical School Animal Management Program, which is accredited by the Association for Assessment and Accreditation of Laboratory Animal Care, International (AAALAC) and meets National Institute of Health standards as set forth in the Guide for the Care and Use of Laboratory Animals (Revised, 2010). The institution also accepts as mandatory the PHS Policy on Humane Care and Use of Laboratory Animals by Awardee Institutions and NIH Principles for the Utilization and Care of Vertebrate Animals Testing, Research, and Training. An Animal Welfare Assurance of Compliance is on file with the Office of Laboratory Animal Welfare (OLAW) (#A3431-01).

### Bacterial strains and plasmids

Msm mc^2^155 (Msm) or Mtb H37Rv were grown at 37°C in Middlebrook 7H9 broth with 0.05% Tween 80 and ADC (0.5% BSA, 0.2% dextrose, 0.085% NaCl, 0.003 g catalase/1L media). Mtb was additionally supplemented with oleic acid (0.006%). For growth curves, overnight cultures were diluted into the appropriate media and growth was either measured by OD_600_ or colony forming units per mL. A summary of all strains, plasmids, and primers used in this study as well as a summary of the construction of conditional mutants can be found in the supporting information (**Text S1**).

### Protein purification and in vitro peptidase assay

The C-terminally 6× His-tagged wild type *clpP1, wild type clpP2, clpP1Ser^98Ala^*, and *clpP2Ser^110Ala^* subunits were overexpressed in Msm using an anhydrotetracycline (ATc) inducible expression system. After overnight induction with ATc (100 ng/mL), cells were lysed by French press, and lysates were centrifuged for 1 h at 100,000 g. The subunits were purified from the supernatant by Ni-NTA affinity chromatography (Qiagen). Eluted fractions containing ClpP proteins were pooled and further purified by size exclusion chromatography on Sephacryl S-300 column. Equal amounts of ClpP1 and ClpP2 (1 µg each) were mixed in the reaction buffer (50 mM K-phosphate buffer pH 7,5, 100 mM KCl, 5% glycerol, 2 mM BME, 5 mM Z-Leu-Leu) and peptidase activity was measured by a rise in fluorescence at 460 nm (Ex at 340 nm) with 0.1 mM Z-Gly-Gly-Leu-AMC as a substrate. To measure dominant negative effect of active site mutants, same reaction was carried out in the presence of 5 µg of the mutant proteins.

### Animal infections

Six to eight week old C57BL/6 mice (Jackson Laboratory) were used for animal infections. Mice were infected via aerosolization with 5×10^6^ CFU each of a 3∶1 mixture of Mtb pTet::ClpP1 S98A and Mtb pTet::GFP (wild type Mtb transformed with a control pTet plasmid containing GFP ). Mice were fed with chow with or without inducer doxycycline. At 30 days after infection, mice were sacrificed, lungs were homogenized and appropriate dilutions were plated on 7H10 plates containing hygromycin or kanamycin to select for the Clp mutant or the control respectively.

## Supporting Information

Figure S1 **PCR confirmation of mycobacterial recombineering.** Primers specific to the 5′-UTR and 3′-UTR of the clpP1P2 operon (RMR13 and RMR16, arrows) were used to distinguish wildtype Msm (expected fragment: 1.8 kb), Msm ptet_ClpP1P2 (expected size: 4.8 kb), and Msm clpP2_ID (expected size 3.2 kb). For each construct, at least one primer was outside homology region used for recombineering in order to ensure specific insertion into the endogenous chromosome.
(TIF)

Figure S2 **Increase in GFP-SsrA upon depletion of ClpP2 is not due to transcriptional activation of GFP-SsrA.** Quantitative PCR of clpP2_ID was carried out to determine if increase in GFP-SsrA was due to transcriptional activation. RNA was isolated from clpP2_ID four hours after induction with ATc (+ATc), and a culture of equal OD_600_ that was left uninduced (−ATc). Using both sigA (left) and 16s rRNA (right) as endogenous controls, there was no significant difference in transcription of GFP-SsrA between induced and uninduced cultures. Data are represented as mean fold change +/− standard deviation, with values normalized to those of the uninduced culture.
(TIF)

Text S1 **Supplementary methods.**
(DOC)
