# Supplementary figures and images for: Mycobacterium tuberculosis ClpP1 and ClpP2 Function Together in Protein Degradation and Are Required for Viability in vitro and During Infection
Source: PLoS Pathog. 2012 Feb 16;8(2):e1002511. doi: 10.1371/journal.ppat.1002511 (PMC3280978; doi:10.1371/journal.ppat.1002511)

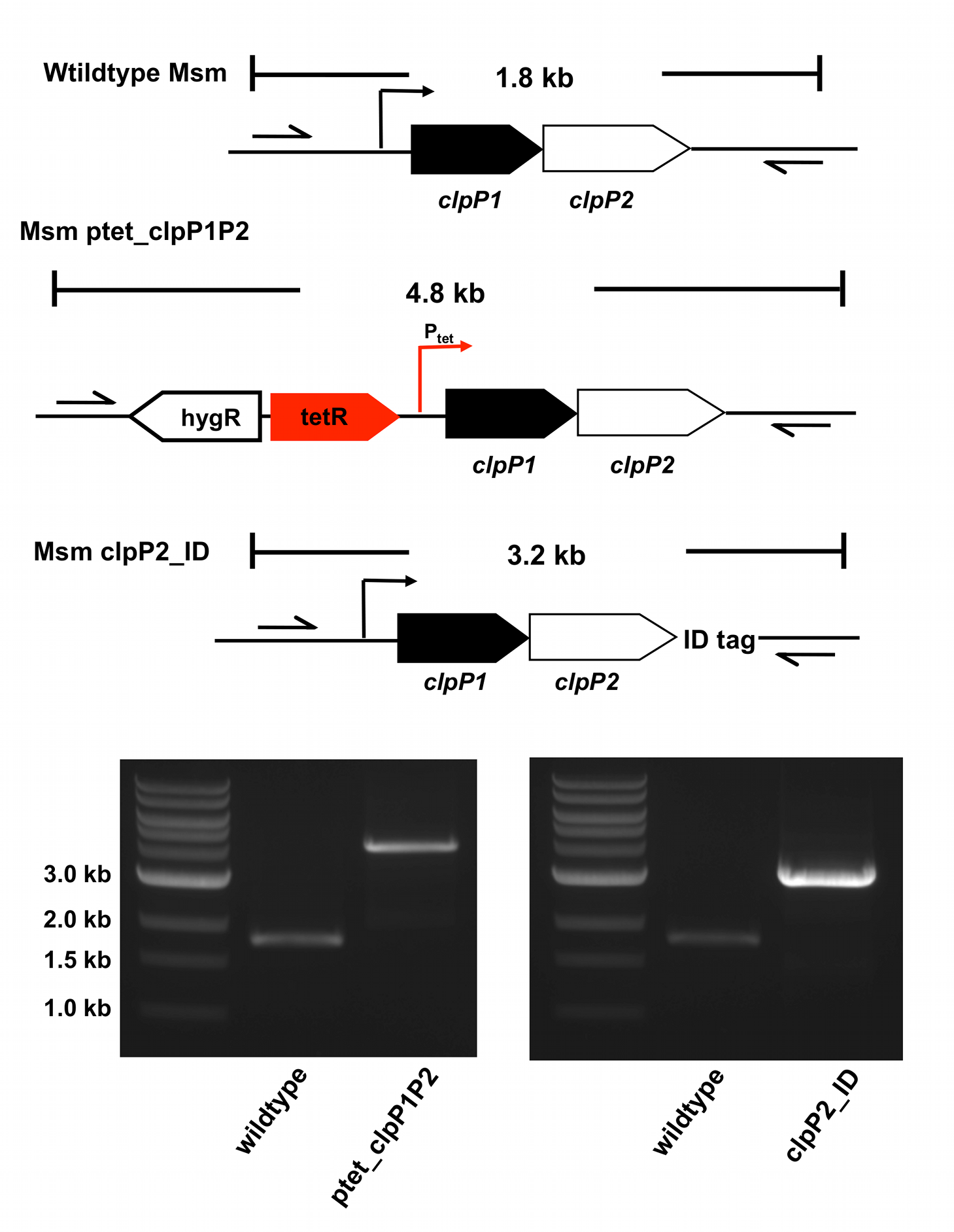

Supplement: Figure S1 — PCR confirmation of mycobacterial recombineering. Primers specific to the 5′-UTR and 3′-UTR of the clpP1P2 operon (RMR13 and RMR16, arrows) were used to distinguish wildtype Msm (expected fragment: 1.8 kb), Msm ptet_ClpP1P2 (expected size: 4.8 kb), and Msm clpP2_ID (expected size 3.2 kb). For each construct, at least one primer was outside homology region used for recombineering in order to ensure specific insertion into the endogenous chromosome. (TIF) [file ppat.1002511.s001.tif]

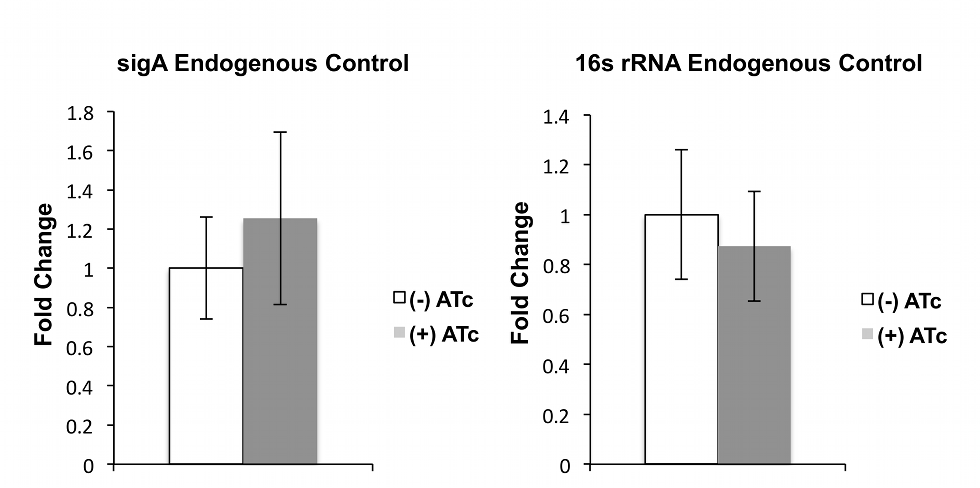

Supplement: Figure S2 — Increase in GFP-SsrA upon depletion of ClpP2 is not due to transcriptional activation of GFP-SsrA. Quantitative PCR of clpP2_ID was carried out to determine if increase in GFP-SsrA was due to transcriptional activation. RNA was isolated from clpP2_ID four hours after induction with ATc (+ATc), and a culture of equal OD600 that was left uninduced (−ATc). Using both sigA (left) and 16s rRNA (right) as endogenous controls, there was no significant difference in transcription of GFP-SsrA between induced and uninduced cultures. Data are represented as mean fold change +/− standard deviation, with values normalized to those of the uninduced culture. (TIF) [file ppat.1002511.s002.tif]
