# Supplementary material for: Mycobacterium tuberculosis ClpP1 and ClpP2 Function Together in Protein Degradation and Are Required for Viability in vitro and During Infection
Source: PLoS Pathog. 2012 Feb 16;8(2):e1002511. doi: 10.1371/journal.ppat.1002511 (PMC3280978; doi:10.1371/journal.ppat.1002511)
Supplement: Text S1 — Supplementary methods. (DOC) [file ppat.1002511.s003.doc]

Supporting Information

**Plasmids**

For complementation studies, wildtype Mtb ClpP1 and ClpP2 were amplified from H37Rv genomic DNA by PCR, using primers RMR01-RMR04 and ligated into the constitutively expressing plasmid pMV762zeo. C terminal 6XHis or c-myc tags were added by PCR primers RMR05-RMR08 on Mtb ClpP1 and ClpP2 and recombined into the ATc inducible vector pTet using gateway recombination (Clontech). Site directed mutagenesis of ClpP1 and ClpP2 was carried out as described previously to generate various catalytic mutants used in the study. Catalytically inactive mutants were inserted into the ATc inducible vector pTet using gateway recombination. Processed Clp mutants were clonted into pTet or pMV762 vectors using primers listed above. The fusion GFP-SsrA was amplified from GFPmut3 wildtype DNA and cloned into pMV762zeo using primers RMR09-RMR12. Details of other plasmids used in this study can be found below.

**Creation of Clp knockdown strains**

Mycobacterial recombineering was employed to create strains ptet_clpP1P2 and clpP2_ID. For strain ptet_clpP1P2, the tetracycline promoter, tetracycline repressor, and a hygromycin resistance marker were inserted into p96863 (Genscript). Both upstream and downstream of the insertion site, p96863 contained 200 bp fragments flanking either side of the native clpP promoter. A linear PCR product containing the regions of homology, the hygromycin resistance marker, and tetracycline repressor and promoter was generated using primers RMR13 and RMR14. Allelic exchange of the native promoter was carried out by transformation of this linear substrate into a Msm strain expressing mycobacteriophage recombinases gp60 and gp61 on a nitrile inducible, counter-selectable episomal plasmid. Counter selection on 10% sucrose led to loss of the recombineering plasmid. Successful integration of the desired sequence was confirmed by PCR, using primers RMR13 and RMR16. As RMR16 lies outside of the homology region used for recombineering, specific integration into the endogenous chromosome could be verified **(Figure S1)**.

To create strain clpP2_ID, a linear DNA substrate was created in a similar fashion. The inducible degradation tag was inserted into p54689 (Genscript). Both upstream and downstream of the insertion site, p54689 contained 200 bp of homology to the C terminal end of clpP2 and the 3’-UTR of clpP2, respectively. A linear PCR product containing this homology and the inducible degradation tag was generated using primers RMR15 and RMR16. This PCR product was transformed into Msm as described above. Successful integration of the desired sequence was confirmed by PCR, using primers RMR13 and RMR16. As RMR13 lies outside of the homology region used for recombineering, specific integration into the endogenous chromosome could be verified **(Figure S1)**.

To make the pTet_clpP2 strain, the tetracycline promoter and ClpP1 were inserted into the suicide plasmid, pSES. Constructs were electroporated into a Msm strain containing an integrated pMC1s vector constitutively expressing the tetR repressor. Integrants were screened by PCR using primers RMR17 and RMR18.

**Inducible degradation of ClpP2**

Mycobacterial recombineering was employed to insert the inducible degradation tag (ID-tag) directly downstream of the clpP2 open reading frame. Inducible degradation was performed as described previously. Briefly, strain clpP2_ID was transformed with an anhydrotetracycline inducible integrated plasmid carrying the HIV-2 protease. Stationary phase cultures were diluted 500-fold, and induced with ATc (50 ng/mL). Cleavage by HIV-2 protease and subsequent protein degradation was monitored by immunoblotting using monoclonal anti-myc (Sigma Aldrich) and monoclonal anti-FLAG (Sigma Aldrich), as the myc and FLAG epitope tags respectively flank the ID-tag. To assess the role of the mycobacterial SsrA-tag, a constitutively expressing plasmid bearing GFP-SsrA was electroporated into clpP2_ID. Cultures were grown and induced as above, and increase in GFP upon HIV-2 protease induction was monitored by fluorescence (emission/excitation: 485/520) and by immunoblotting using monoclonal anti-GFP (Invitrogen). For all immunoblotting, total protein lysates were prepared from equivalent cell numbers using bead beating. After probing with primary antibody, visualization was performed using HRP-conjugated secondary antibodies, and detection was performed using SuperSignal West Femto Chemiluminescent Substrate (ThermoScientific) according the manufacturer’s protocol. In all cases, blots were stripped and reprobed with monoclonal anti-RpoB (MyBioSource) to ensure equivalent loading of samples.

**Table of main plasmids used in this study:**

| **Plasmid** | **Properties/Uses** |
| --- | --- |
| **pTetOR::clpP1-myc** | Inducible expression of c-myc-tagged Mtb ClpP1 (to assess in vivo interaction with ClpP2) |
| **pTetOR::clpP2-his** | Inducible expression of 6xHis-tagged Mtb ClpP2 (to assess in vivo interaction with ClpP1) |
| **pTetOR::clpP1wt** | Inducible expression of Mtb ClpP1 (for in vitro degradation assay) |
| **pTetOR::clpP1S** | Inducible expression of Mtb ClpP1-Ser^98A^ (for in vitro degradation assay, and overexpression in Mtb) |
| **pTetOR::clpP2S** | Inducible expression of Mtb ClpP2-Ser^110A^ (for in vitro degradation assay, and overexpression in Mtb) |
| **pTetOR::clpP1H** | Inducible expression of Mtb ClpP1-His^123A^ (for overexpression in Mtb) |
| **p96863** | Non-expressing, synthesized plasmid (Genscript) containing regions of homology to ClpP1 5’UTR and ORF. Used to generate linear PCR product for recombineering to create Msm ptet_clpP1P2 |
| **pKM339** | Plasmid used to obtain tetracycline promoter, repressor, and hygromycin resistance marker, which were cut and inserted into p96863 for recombineering to create Msm ptet_clpP1P2 |
| **p54689** | Non-expressing, synthesized plasmid (Genscript) containing regions of homology to ClpP2 ORF and 3’UTR. Used to generate linear PCR product for recombineering to create Msm clpP2_ID |
| **puc57::inhA-ID** | Plasmid used to obtain inducible degradation tag, which was cut and inserted into p54869 for recombineering to create Msm clpP2_ID |
| **pSES::ptet_clpP1** | Plasmid used for homologous recombination to create Msm ptet_clpP2 |
| **pMV762zeo::clpP1** | Constitutively expressing plasmid expressing Mtb ClpP1 for complementation studies |
| **pMV762zeo::clpP2** | Constitutively expressing plasmid expressing Mtb ClpP2 for complementation studies |
| **pMV762zeo::clpP1P2** | Constitutively expressing plasmid expressing the entire clpP1clpP2 operon for complementation studies |
| **pMV762zeo::GFP-SsrA** | Constitutively expressing plasmid expressing the fusion construct GFP-SsrA to assess role of Clp protease in degradation of SsrA-tagged substrates |
| **pSES::ClpP1** | Plasmid used for homologous recombination event to create strain, pTet_clpP2 |
| **pNit::Che9c** | Plasmid expressing mycobacteriophage recombinases, used in mycobacterial recombineering |

**Table of main primers used in this study:**

| **Primer Name** | **Primer Sequence (5’ to 3’)** | **Use** |
| --- | --- | --- |
| **RMR01** | GCACTGTTAATTAAGAAGGAGATATACCTATGCGTTCGAACTCGCAG | Cloning of processed Mtb ClpP1 into pTetOR, pmv (forward) |
| **RMR02** | AGTATACAGCTGTCACTGTGCTTCTCCATTGACCTG | Cloning of processed Mtb ClpP1 into pTetOR (reverse) |
| **sRMR03** | GCACTGTTAATTAAGAAGGAGATATACCTATG CGCTACATCCTGCCGTC | Cloning of processed Mtb ClpP2 into pTetOR, pmv (forward) |
| **RMR04** | AGTATACAGCTGTCAGGCGGTTTGCGCGGA | Cloning of processed Mtb ClpP2 into pTetOR, pmv (reverse) |
| **RMR05** | AGTATACAGCTGTCACAGGTCCTCCTCCGAGATCAGCTTCTGCTCCTGTGCTTCTCCATTGACCTG | Cloning of processed Mtb ClpP1-myc into pTetOR (reverse) |
| **RMR06** | AGTATACAGCTGGTGGTGGTGGTGGTGGTGCTGTGCTTCTCCATTGACCTG | Cloning of processed Mtb ClpP1-his into pTetOR (reverse) |
| **RMR07** | AGTATACAGCTGTCACAGGTCCTCCTCCGAGATCAGCTTCTGCTCGGCGGTTTGCGCGGA | Cloning of processed Mtb ClpP2-myc into pTetOR, pmv (reverse) |
| **RMR08** | AGTATACAGCTGGTGGTGGTGGTGGTGGTGGGCGGTTTGCGCGGA | Cloning of processed Mtb ClpP2-his into pTetOR, pmv (reverse) |
| **RMR09** | GATCCGCATGCTTAATTAAGAAGGAG | Cloning of GFP-ssrA into pmv (forward) |
| **RMR10** | GTGGTGGTGATGGATGGTGTTTGTATAGTTCATCCATGCCATG | Cloning of GFP-ssrA into pmv (reverse, first round) |
| **RMR11** | CTGATGTGAATCGGCGTGGTGGTGATGATGGTGTTTCTATAG | Cloning of GFP-ssrA into pmv (reverse, second round to add first portion SsrA-tag) |
| **RMR12** | CGGAATATCGATCTAGGCAGCGAGAGCGTAGTCGCGCTGATGTGAATCGGC | Cloning of GFP-ssrA into pmv (reverse, third round to add remaining portion SsrA-tag) |
| **RMR13** | CCGCCGTGGCCTGACCATC | Generation of linear PCR product from p96863 to recombineer strain ptet_clpP1P2 (forward) |
| **RMR14** | TCTTCCGCCGACAGCAACAGG | Generation of linear PCR product from p96863 to recombineer strain ptet_clpP1P2 (reverse) |
| **RMR15** | CATCCAGGGCCAGTTCTC | Generation of linear PCR product from p54689 to recombineer strain clpP2_ID (forward) |
| **RMR16** | CGTGGTGTTTGCCGTTCT | Generation of linear PCR product from p54689 to recombineer strain clpP2_ID (reverse) |
| **RMR17** | GCACGGCATACATCATTTCGACGCCG | Used in screening for pTet_clpP2 strain (forward). Binds to the tetracycline promoter. |
| **RMR18** | GGCGGTTTGCGCGGAGAGC | Used in screening for pTet_clpP2 strain (reverse). Binds to the 3’-end of clpP2. |
